# The Use of Chitin for the Removal of Nitrates and Orthophosphates from Greenhouse Wastewater

**DOI:** 10.3390/molecules29061289

**Published:** 2024-03-14

**Authors:** Tomasz Jóźwiak, Artur Mielcarek, Urszula Filipkowska

**Affiliations:** Department of Environmental Engineering, University of Warmia and Mazury in Olsztyn, Warszawska St. 117a, 10-957 Olsztyn, Poland; artur.mielcarek@uwm.edu.pl (A.M.); urszula.filipkowska@uwm.edu.pl (U.F.)

**Keywords:** chitin flakes, adsorbent, unconventional sorbent, sorption, greenhouse wastewater, nutrients, phosphates, nitrates, precipitation

## Abstract

The study investigated the possibility of using chitin flakes as an unconventional sorbent for the removal of orthophosphates and nitrates from greenhouse wastewater (GW). The effluent parameters were as follows: 66.2 mg P-PO_4_/L, 566.0 mg N-NO_3_/L, 456.0 mg S-SO_4_/L, 13.7 mg Cl^−^/L, 721 mg Ca^2+^/L, 230 mg Mg^2+^/L, hardness 11.3 °dH, and pH 5.4. The scope of the research included determinations of the influence of pH on GW composition and the efficiency of nutrient sorption, the kinetics of nutrient sorption, the influence of the dose of chitin flakes on the effectiveness of nutrient binding and the maximum sorption capacity of the sorbent. The sorption of P-PO_4_ on the tested sorbent was most effective at pH 4, and the sorption of N-NO_3_ at pH 2. The equilibrium time of sorption of both nutrients from GW to chitin depended on the sorbent dose and ranged from 150 to 180 min. The sorbent dose of 40 g/L enabled removing 90% of orthophosphates and 5.7% of nitrates from the wastewater. The maximum sorption capacity of CH towards P-PO_4_ and N-NO_3_ contained in the GW was 3.20 mg/g and 3.04 mg/g, respectively. In turn, the sorption of calcium and magnesium ions on chitin flakes was completely ineffective.

## 1. Introduction

Greenhouse gardening, entailing soilless cultivation, is widely recognized as a modern form of agriculture. It allows for strict control of the process and saving water for irrigation of plants, regardless of weather conditions [1]. In this system, the water solution of minerals necessary for crops is delivered directly to the root zone of the plants. In order to ensure maximum nutrient uptake, the media often contain excessive doses [2]. For this reason, greenhouse wastewater (GW) generated after plant irrigation contains significant amounts of unused minerals, such as phosphates, nitrates, sulfates, calcium, and magnesium [3].

Greenhouse wastewater (GW) has a very low organic carbon content, which prevents it from being putrefacted. It is, therefore, suitable for storage, and after being supplemented with the missing amounts of nutrients, it may be used again as a nutrient medium for plants [4]. However, wastewater generated in greenhouses may contain disease-causing pathogens that can harm crops. Hence, this cultivation method poses the risk of significant yield losses [5]. The problem of the presence of pathogens in the wastewater-based culture medium can be solved by its disinfection, which is, however, very expensive [6]. For this reason, most producers do not recycle greenhouse wastewater and discharge it into the sewage system or directly into the soil without pre-treatment [7].

Due to the significant concentrations of phosphates and nitrates released into the environment, GW can contribute to the eutrophication of local water reservoirs and also pollute groundwater [8]. Discharging GW into the sewage system is also not an optimal solution. In turn, discharging high loads of nitrogen and phosphorus into a conventional biological treatment plant with relatively small amounts of organic carbon can significantly hamper its operation.

Therefore, it seems reasonable to pre-treat the GW at the point of origin. The technology used should focus on the removal of nutrients (nitrates and phosphates) from the wastewater. Precipitation methods based on the use of coagulants, such as calcium hydroxide, aluminum or iron sulfate, prove effective in removing phosphorus from aqueous solutions [9]. However, their major disadvantage is the large amounts of precipitated sludge that are produced during the coagulation process and are difficult to manage [10]. Another problem with the use of coagulation is the increasing price of coagulants. Other proven methods for removing nitrates from water and wastewater include electrodialysis [11] and reverse osmosis [12]; however, they are still expensive.

An alternative way to remove both nitrates and phosphates from GW is to sorb them. The effectiveness and price of sorption depend largely on the type of sorbent used. So far, the ability to sorb nutrients has been tested on such sorption materials as biochar [13,14], activated carbon [15,16], ion exchange resins [17,18], plant waste biomass [19,20], and chitosan materials [21,22]. Chitosan-based materials proved to be one of the most efficient sorbents for nitrates and phosphates. The sorption capacity of properly prepared chitosan sorbents can reach >100 mg/g for nitrate ions and >300 mg/g [23] for orthophosphate ions.

Chitosan is a polysaccharide, a deacetylated form of chitin, which is the main building block of the exoskeleton of arthropods [24]. Its high sorption capacity towards nitrates and phosphates is due to its amine functional groups. These groups can be easily protonated in a broad pH range, imparting a basic character to chitosan [21].

Chitosan-NH_2_ + H_3_O^+^ → Chitosan-NH_3_^+^ + H_2_O

The positively charged amino groups on the chitosan surface are able to attract nitrate and orthophosphate anions electrostatically, which considerably increases the efficiency of their sorption. Chitosan sorbents have already proven their high usefulness in removing nutrients from greenhouse effluents [25]; however, they are relatively expensive, partly due to the high production costs. Chitin, a precursor of chitosan, is a much cheaper sorbent material. It differs from chitosan in that it has functional acetamide groups instead of amino groups. Its acetamide groups can also be protonated [26], which makes them potentially active sites for nitrate and orthophosphate ions. Chitin-based materials can, therefore, offer a reasonable alternative to chitosan sorbents. The most common and cheapest on the market is chitin from snow crab shells in the form of flakes.

The present study investigated the possibility of using chitin flakes to remove orthophosphates and nitrates from the GW. The scope of the research included determinations of the influence of pH on the efficiency of removal of the main components from wastewater (P-PO_4_; N-NO_3_; S-SO_4_; Ca^2+^; Mg^2+^); nutrient sorption kinetics; and the maximum sorption capacity of chitin towards orthophosphates and nitrates.

## 2. Results and Discussion

### 2.1. The Influence of pH on GW Composition and the Efficiency of Nutrient Sorption on CH

GW is a mixture containing nitrates, orthophosphates, and other components, such as chlorides, sulfates, and calcium and magnesium ions. As is generally known, Ca^2+^ and Mg^2+^ cations can be precipitated with phosphate or sulfate anions. The effectiveness of precipitation of calcium and magnesium deposits depends mainly on the molar ratio between cations and anions and on solution pH. When treating GW via sorption, some of the phosphates may be removed by precipitation. To reduce the risk of incorrect data analysis, the influence of the pH value itself on the concentration of individual GW components was investigated first.

**Figure 1:**
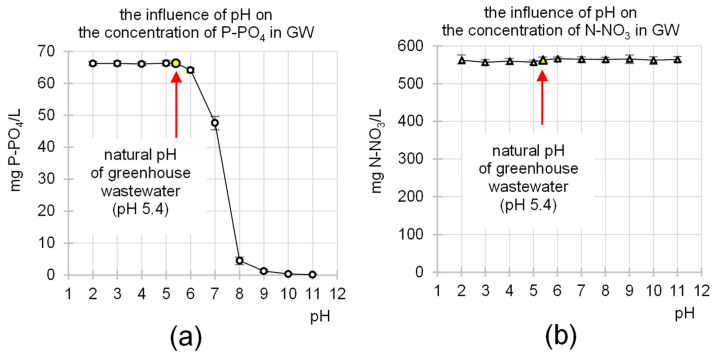
The influence of pH on the concentration of (**a**) P-PO_4_ and (**b**) N-NO_3_ in GW. Reaction time—60 min; temp. 20 °C.

The natural pH value of GW was pH 5.4. Acidification of the wastewater with HCl had no significant effect on the orthophosphate concentration (Figure 1a). In turn, the concentration of P-PO_4_ in GW decreased with increasing pH during its alkalization with NaOH. Wastewater pH correction to pH 7 caused a 28% decrease in the concentration of orthophosphates, while pH correction to pH 8 and pH 9—reduced it by 93 and 98%, respectively. An almost complete removal of phosphorus from the wastewater (>99.8%) was achieved by alkalizing the sewage sludge to pH > 10.

**Figure 2:**
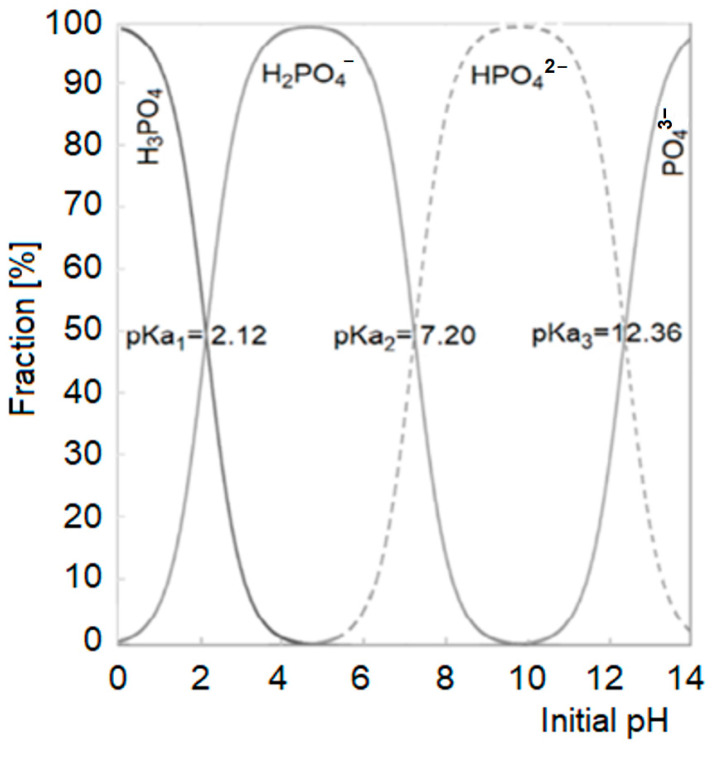
The effect of pH on the form of orthophosphate ions in the solution. Based on [27].

As already mentioned, the removal of orthophosphates from GW, initiated by its alkalization with NaOH, occurred via precipitation with calcium and magnesium ions. The nature of the precipitated phosphate salts largely depended on the form of the orthophosphate ion (Figure 2).

Due to the variety of possible forms of phosphorus compounds precipitated with calcium and magnesium (hydrogen phosphates, hydroxyapatites), it is difficult to clearly determine the exact pattern of the reactions taking place and the chemical formula of the salts obtained. However, the phosphate precipitation reactions proceeding in the GW can be described using a simplified model.

- In the pH range of 5.4–7.2 (most orthophosphates are in the form of the H_2_PO_4_^−^ anion)
  - 2 H_2_PO_4_^−^ + Ca^2+^ → Ca(H_2_PO_4_)_2_ (solubility in water ~20 g/L at 20 °C);
  - 2 H_2_PO_4_^−^ + Mg^2+^ → Mg(H_2_PO_4_)_2_ (solubility in water ~200 g/L at 20 °C).
- In the pH range of 7.2–11.0 (most orthophosphates are in the form of the HPO_4_^2−^ anion)
  - HPO_4_^2−^ + Ca^2+^ → CaHPO_4_↓ (solubility in water ~0.1 g/L at 20 °C);
  - HPO_4_^2−^ + Mg^2+^ → MgHPO_4_↓ (solubility in water ~0.25 g/L at 20 °C).

Due to the inability to precipitate nitrates with other wastewater components, the pH correction in the system had no major influence on the N-NO_3_ concentration in the GW (Figure 1b).

**Figure 3:**
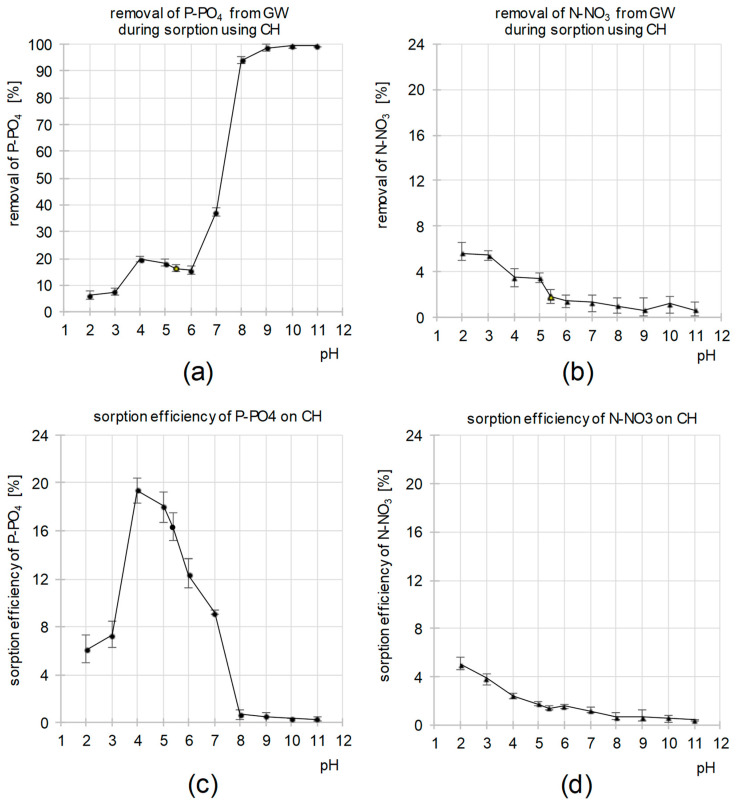
The influence of pH on the removal efficiency of (**a**) P-PO_4_ and (**b**) N-NO_3_ from GW as a result of sorption and precipitation. The influence of pH on the removal efficiency of (**c**) P-PO_4_ and (**d**) N-NO_3_ from GW as a result of only sorption. Sorbent dose—5 g/L; sorption time—60 min; temp. 20 °C.

Figure 3a,b show the efficiency of P-PO_4_ and N-NO_3_ removal from the system during sorption at different initial pH values of the GW. The efficiency of eliminating orthophosphate ions generally increased with increasing pH of the wastewater. The largest increase in P-PO_4_ removal efficiency (from 16 to 94%) was recorded in the pH range of 6–8 (Figure 3a).

Considering the previous experiment, the results of which are shown in (Figure 1a), it can be stated with great certainty that, in the pH range of 7–11, the removal of P-PO_4_ from the system occurs mainly via precipitation of orthophosphates with calcium and magnesium. This is also confirmed by our previous studies on the sorption of P-PO_4_ on CH from distilled water-based solutions [28], according to which the sorption intensity of orthophosphate ions is low at pH > 7.

After taking into account the amounts of orthophosphates removed from the system via precipitation with calcium and magnesium ions, the theoretical efficiency of P-PO_4_ sorption onto CH was calculated as a function of pH (Figure 3c). According to the calculations, the binding efficiency of orthophosphates to CH was the highest at pH 4 and decreased with increasing pH. In the pH range of 8–11, the efficiency of P-PO_4_ removal from the system via sorption was <1%. A significant decrease in the sorption efficiency of this nutrient to CH was also observed at pH < 4 (Figure 3c).

The efficiency of N-NO_3_ removal from GW was the highest at pH 2 and decreased with increasing pH of GW (Figure 3b). Due to the inability to precipitate N-NO_3_ with the cations present in the wastewater, it can be assumed that the elimination of nitrate ions from the system occurred via sorption onto CH. This is also confirmed by the close similarity between the data shown in Figure 3b,d.

The relatively high efficiency of nutrient sorption to CH at acidic pH was the result of the ability to protonate the nitrogen atom in the acetamide functional groups in the presence of excess hydronium ions in the system.

Chitin-NH-CO-CH_3_ + H_3_O^+^ → Chitin-NH_2_^+^-CO-CH_3_ + H_2_O

Positively charged functional groups attracted orthophosphate and nitrate anions electrostatically, which increased the efficiency of their sorption. With increasing pH, the concentration of hydronium ions decreased, which reduced the efficiency of protonation of the acetamide groups and, consequently, the efficiency of anion sorption.

The positive effect of low pH on the efficiency of P-PO_4_ sorption was also observed in a study on the removal of orthophosphates on chitosan-based sorbents [29,30]. In turn, a high nitrate binding efficiency at low pH was observed for sorbents such as clay [31], activated carbon [32,33], and *Dioscorea alata* L. biomass [34].

The decrease in sorption efficiency of P-PO_4_ on CH at pH < 4 could be due to the relatively large amounts of orthophosphates in the H_3_PO_4_ form (non-ionized) (Figure 2), which were unable to interact electrostatically with the protonated functional groups of the sorbent. A similar result was also obtained in our previous study addressing the sorption of phosphates onto chitin and chitosan sorbents from distilled water-based solutions [23,30]. The reduced efficiency of phosphate binding to CH at pH ≤ 3 could also be influenced by the increasing competition with chlorides for free sorption centers (the pH was corrected by dosing HCl).

**Figure 4:**
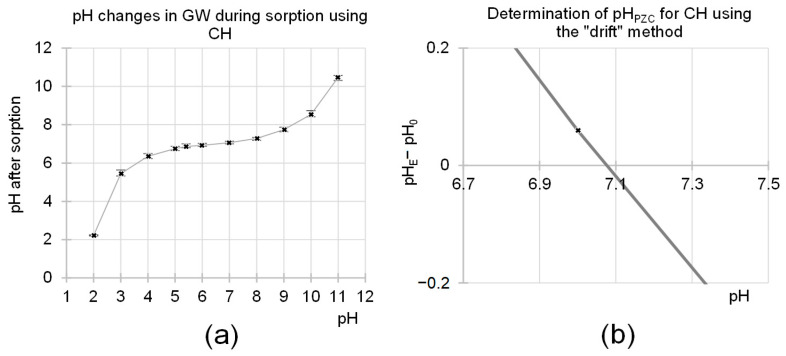
(**a**) The effect of CH on changes in the pH of GW during sorption. (**b**) Determination of the pH_PZC_ of CH using the “drift” method. On the *Y*-axis: pH_E_—the pH after 24 h of contact of the sorbent with the solution; pH_0_—the initial pH. Sorbent dose—5 g/L; sorption time—60 min; time of determination of pH_PZC_—240 min; temp. 20 °C.

CH was observed to modify the pH of GW. In the initial wastewater pH range of pH 4–9, the pH value after sorption was between pH 6.6 and pH 7.7 (Figure 4a). This result is because CH has functional groups capable of ionization. The system always strives for a pH value close to the pH_PZC_ value (PZC—Point of Zero Charge), which, when determined for CH with the “drift” method, was pH_PZC_ = 7.1 (Figure 4b). This confirms the slightly basic nature of CH and results from its acetamide functional groups.

**Figure 5:**
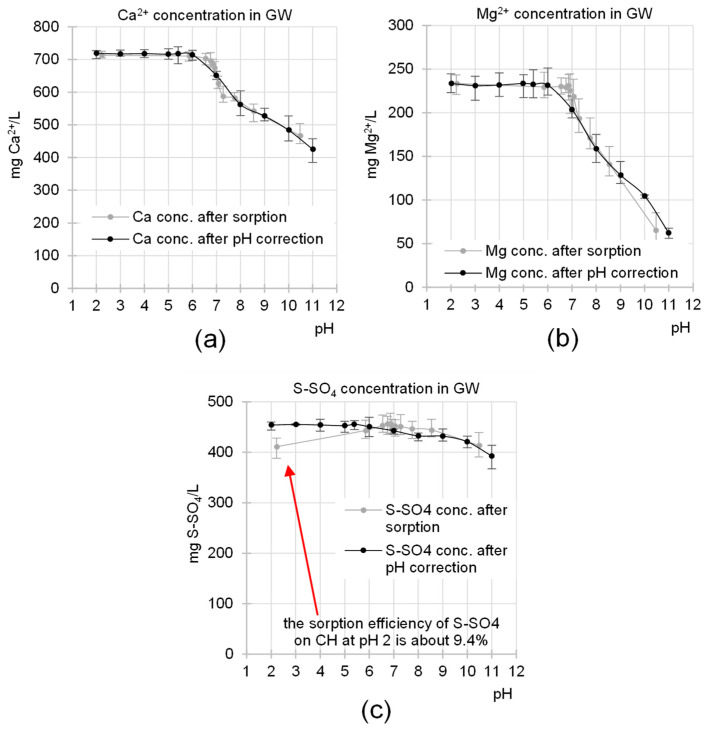
Correlation between the pH value after sorption/pH correction and the concentration of (**a**) Ca^2+^, (**b**) Mg^2+^, and (**c**) S-SO_4_ in the GW. Sorbent dose 5 g/L (in experimental series with sorbent); sorption/reaction time—60 min; temp. 20 °C.

The influence of the sorption pH value on the concentration of other important GW components (Ca, Mg and S-SO_4_) was also investigated. To reduce the risk of data misinterpretation, the results obtained were compared with the effects of pH correction alone on the concentration of these components in the wastewater (Figure 5).

Correcting the pH value of GW above its natural pH value (pH 5.4) significantly affected the concentrations of calcium and magnesium ions, which decreased along with increasing pH (Figure 5a,b). The same trend was observed for sulfate ions (Figure 5c). The removal of these ions from the system, triggered by pH correction, resulted from the coprecipitation of orthophosphate and sulfate anions with calcium and magnesium cations (in the case of SO_4_^2−^ only with Ca^2+^). At higher pH (>9), some Ca^2+^ and Mg^2+^ ions were precipitated as hydroxides. The amounts of precipitated ions increased with increasing pH, which explains why the concentration of Ca^2+^ and Mg^2+^ in the system continued to decrease despite the limited amount of orthophosphate ions (Figure 5a,b). Correcting the pH of GW below its natural pH (pH 5.4) had no effect on the concentrations of calcium or magnesium ions.

The diagrams shown in Figure 5 contain experimental data from studies on the influence of the sorption process on the concentrations of calcium, magnesium and sulfates in GW. Due to the influence of CH on the change in wastewater pH during sorption and the significant influence of pH itself on the concentrations of these components in GW, we have decided to plot the concentration of wastewater components as a function of the final pH of the wastewater (pH after sorption). This procedure was expected to show the actual differences between the elimination of these ions via precipitation (caused by the pH correction) and their removal from the solution via sorption to CH. It turned out that the experimental data from studies on the effects of pH correction on the concentration of calcium and magnesium in the sludge were consistent with the results of experiments on their sorption at different pH values of the wastewater. This indicates that the removal of calcium and magnesium ions from wastewater during the sorption process was practically only a consequence of their precipitation.

Analyses of the removal of sulfates from GW demonstrated a significant decrease in S-SO_4_ concentration (9.4%) during sorption at pH 2. Such a decrease in sulfate concentration was not observed when analyzing the effects of pH correction itself on the effluent composition (Figure 5c). This proves that this load of sulfates was removed from the system via sorption.

The next steps of the research on the removal of nutrients from GW via sorption were carried out at pH 4, the most favorable pH for the sorption of P-PO_4_ from wastewater. This decision was made due to the great economic importance of phosphorus and the fact that its concentration in natural waters is a factor minimizing the eutrophication process.

### 2.2. Kinetics of the Sorption of Nutrients onto CH

**Figure 6:**
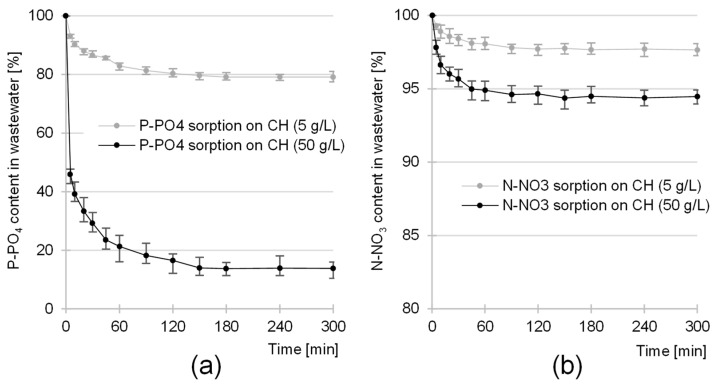
Changes in the concentration of (**a**) P-PO_4_ and (**b**) N-NO_3_ in GW during sorption using CH as a sorbent. Sorbent dose—5/50 g/L; initial pH 4; temp. 20 °C.

**Figure 7:**
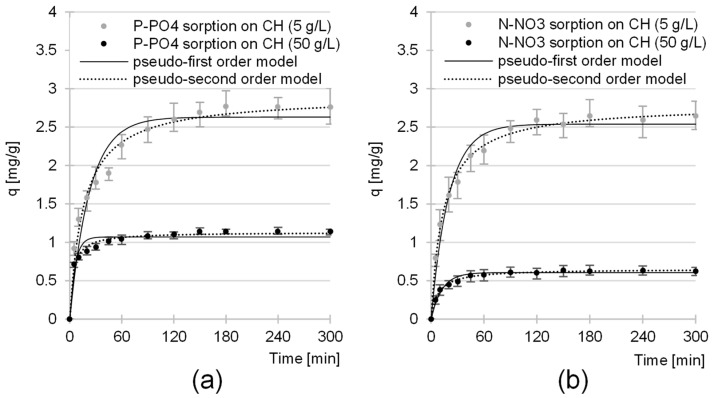
Sorption kinetics of (**a**) P-PO_4_ and (**b**) N-NO_3_ from GW to CH. Pseudo-first-order and pseudo-second-order models. Sorbent dose—5/50 g/L; initial pH 4; temp. 20 °C.

The equilibrium time of the sorption of nutrients (orthophosphates and nitrates) to CH from GW depended on the sorbent dose and was 180 min for 5 g/L and 150 min for 50 g/L (Figure 6a,b). The sorption efficiency of these components was the highest at the beginning of the process. After only 20 min, the amount of orthophosphates bound to CH was between 57 and 77% of the *q_e_* value, and, in the case of nitrates, it was between 61 and 72% of the *q_e_* value (Figure 7a,b).

Similar equilibrium times for phosphate sorption were determined during the treatment of aqueous solutions with chitosan hydrogels (120 min) [30] and activated carbon fibers (180 min) [35]. For nitrates, similar sorption equilibrium times were recorded during nutrient removal by means of granular activated carbon (120 min) [36], cross-linked chitosan (120 min) [37], and hydrotalcite (120 min) [38].

The shorter nutrient sorption times in the case of the higher sorbent dose are probably due to the higher probability of collisions of the sorbate anions with the CH sorption centers.

Experimental data from studies on the kinetics of P-PO_4_ and N-NO_3_ sorption from GW were described with pseudo-first and pseudo-second-order models (Figure 7, Table 1). In each analytical series, regardless of CH dose, the pseudo-second-order model showed the best fit to the data, which is a typical result in studies on the sorption of nutrients to biosorbents.

As expected, the rate of nutrient removal from the system was greater at higher CH doses (Figure 7). However, the values of the *k_2_* and *q_e_* constants determined using the pseudo-second-order model show that as the sorbent dose increased and the efficiency per unit mass decreased, which was probably due to a significant increase in the ratio of CH sorption centers to the nutrient anions available in the solution.

**Figure 8:**
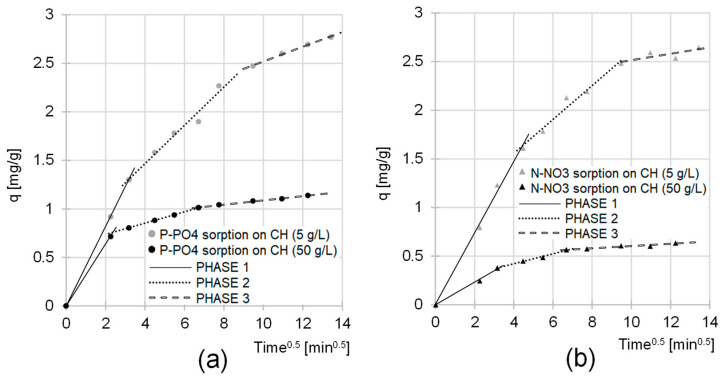
Intraparticle diffusion model of (**a**) P-PO_4_ and (**b**) N-NO_3_ sorption from GW to CH. Sorbent dose—5/50 g/L; initial pH 4; temp. 20 °C.

The sorption of nutrients to CH was also described by the intraparticle diffusion model (Figure 8, Table 2). The data obtained indicate that this process occurs in three main phases, which differ in intensity and duration. In the first, shortest, but most intense phase, orthophosphate and nitrate ions occupied the most accessible CH sorption centers on the surface of the flocs. The second phase began when most of the active sites on the chitin surface were saturated. In this phase, the sorbate anions began to bind to the active sites in the macro- and mesopores of the chitin flakes. Due to the limited availability of sorption sites and the strong competition between sorbate ions, the second sorption phase was characterized by a much lower intensity than the first phase and by a longer duration. After most of the active sites available in the structure of the CH flakes were occupied, the third phase began. It involved the slow binding of sorbate ions to the most difficult-to-reach sorption centers, such as those located in micropores. The third phase of sorption ended when sorption equilibrium had been reached in the system.

The *k_d1_*, *k_d2_* and k_d3_ constants determined from the model had lower values in the analytical series with the higher CH doses (Table 2). As already mentioned in this section, this can be explained by a much smaller ratio of the amount of nutrients to the available active centers in the system. In turn, the shorter duration of the main sorption phases (first and second phase) in the case of higher CH doses results from a higher probability of collisions of the sorbates with the sorbent sorption centers and, therefore, a faster saturation of its active sites.

**Figure 9:**
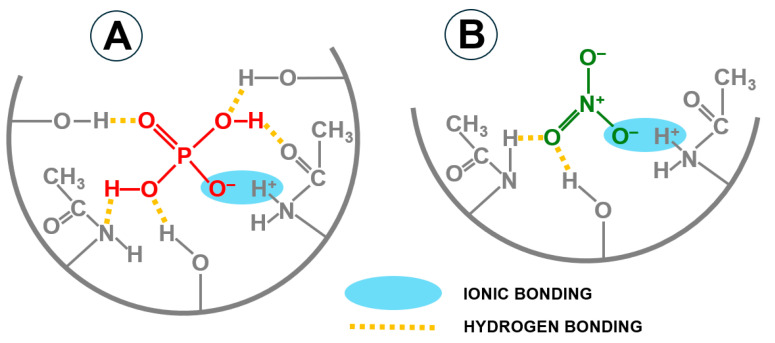
Probable modes of nutrient bonding (pH 4–7) onto the chitin chain: (**A**) orthophosphates and (**B**) nitrates.

The *q_e_* values determined for the nutrients (pseudo-second-order model) and *k_d1_*, *k_d2_ k_d3_* (intraparticle diffusion model) indicate a higher sorption performance of orthophosphates on CH than nitrates. It is important to note that the preferential sorption of phosphates by the tested sorbent occurs despite the multiple higher concentrations of nitrates in the wastewater. At pH 4, the ions of these nutrients are generally present in the form of H_2_PO_4_^−^ and NO_3_^−^. The higher binding efficiency of orthophosphates to CH compared to nitrates could be due to the possibility of forming more hydrogen bonds between sorbate and chitin functional groups (acetamide, amino and hydroxyl groups) (Figure 9).

### 2.3. Influence of Sorbent Dose on the Efficiency of P-PO_4_ and N-NO_3_ Sorption from GW

**Figure 10:**
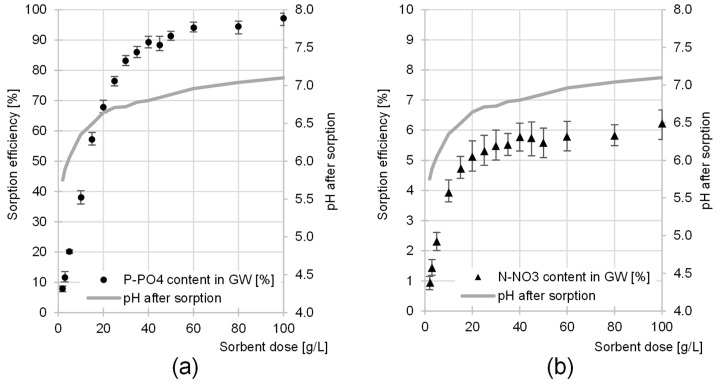
The effect of CH dose on the sorption efficiency of (**a**) P-PO_4_ and (**b**) N-NO_3_ from GW. Initial pH of sorption—pH 4; temp. 20 °C.

The efficiency of nutrient removal from GW increased with an increasing sorbent dose (Figure 10). The orthophosphate sorption efficiency of 90% was achieved at a CH dose of 40 g/L. As the CH dose was further increased, the increase in sorption efficiency was small. At a CH dose of 100 g/L, the efficiency of P-PO_4_ removal from GW was 97% (Figure 10a).

The strongest correlation between CH dose and nitrate sorption efficiency was determined up to a CH dose of 20 g/L, at which N-NO_3_ sorption efficiency was 5.1% (Figure 10b). A further increase in the sorbent dose did not effectively increase nitrate removal efficiency from the GW. With five times the amount of sorbent used (100 g/L), the removal rate of this nutrient increased by only 1.2% (to 6.2%) (Figure 10b).

A very small increase in the efficiency of nitrate sorption with an increasing sorbent dose, determined at CH doses of 20–100 g/L (Figure 10b), was probably due to changes in pH caused by the presence of chitin in the GW. The initial pH of the effluent was pH 4. As the sorbent dose was increased, the final pH of the effluent increasingly approached the pH_PZC_ value typical of CH (pH_PZC_ = 7.1). As explained in Section 2.1, an increase in pH in the system can lead to a significant reduction in the sorption capacity of nitrates on CH. At pH > 6, the sorption efficiency of this nutrient is very low, which could explain the very small increase in N-NO_3_ sorption efficiency with an increasing CH dose > 20 g/L (Figure 10b). A similar result was not observed in the case of orthophosphates, as the sorption efficiency of these nutrients is relatively high in the pH range of 4–7, and their concentration in wastewater is much lower.

#### Maximum Nutrient Sorption Capacity of CH

**Figure 11:**
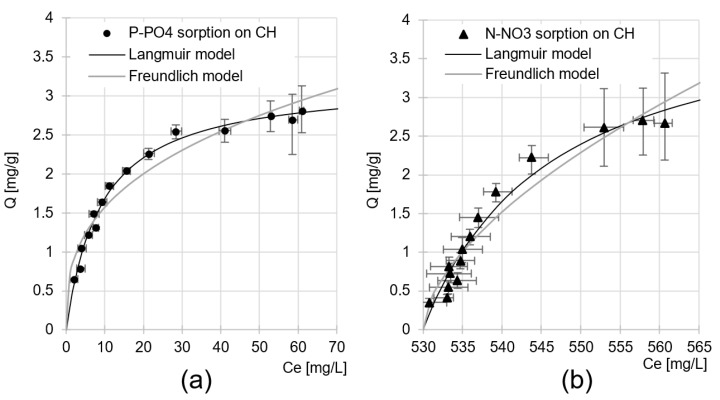
Isotherms of sorption of (**a**) P-PO_4_ and (**b**) N-NO_3_ on CH from GW. Initial pH 4; sorption time—180 min, temp. 20 °C.

The maximum sorption capacity of CH was determined based on data from analyses on the influence of sorbent dose on the efficiency of nutrient sorption from GW. The experimental data were described using Langmuir and Freundlich isotherms (Figure 11). In all analytical series, nutrient sorption to CH was best described by the Langmuir isotherm. This model assumes that only one sorbate particle can be bound to a sorption center at a time, but the bond formed between them is unstable. Nutrients bound to CH form the so-called “monolayer” on the surface of the sorbent in which they can move.

The maximum sorption capacity of CH towards the nutrients present in the GW was Q_max_ = 3.20 mg P-PO_4_/L for orthophosphates and *Q_max_* = 3.04 mg N-NO_3_/g for nitrates (Table 3). Considering the values of the constants determined from the Langmuir model (*Q_max_/K_C_*) and the initial concentrations of the nutrients in the wastewater (66.20 mg P-PO_4_/L; 566.00 mg NO_3_/L), it can be confirmed with certainty that orthophosphate ions have a much greater affinity for the CH sorption centers than nitrate ions. As mentioned in Section 2.2, this could be due to the structure of the orthophosphate ion, which allows the formation of more hydrogen bonds with chitin functional groups compared to nitrate ions (Figure 9). Preferential binding of orthophosphates from a mixture containing nitrates was also observed in our previous study on nutrient sorption to chitosan sorbents [23,29].

Table 4 compares the sorption efficiency of nutrients from wastewater on different sorbents. The sorption efficiency of orthophosphates and nitrates on chitin flakes is relatively low. Compared to CH, the sorption of nutrients from greenhouse wastewater on chitosan sorbents is much more efficient. For example, the sorption efficiency of orthophosphates and nitrates on chitosan hydrogel beads cross-linked with epichlorohydrin can be almost 10 times higher than on CH. However, it should be borne in mind that chitosan sorbents are much more expensive to produce than chitin flakes. Aminated sorbents, whose surface is enriched with primary amine groups, also have a higher sorption capacity than chitin flakes. This could indicate orthophosphates and nitrates have a much greater affinity for protonated amine functional groups than ionized acetamide groups. This is probably due to differences in the structure of the two functional groups. The acetyl group, which is part of the acetamide group of chitin, can significantly restrict access to the local positive charge (generated by the proton bound to the nitrogen). This can lead to weaker electrostatic interactions of nutrients with the basic functional groups of chitin and consequently lower sorption efficiency.

It is worth noting that the suitability of chitin flakes for the sorption of nutrients from wastewater is greater than that of unconventional sorbents, such as fly ash, slag or plant biomass (Table 4).

The literature data collected in Table 4 indicate that preferential sorption of orthophosphates in the nutrient mixture occurs not only on chitin and chitosan sorbents but also on biochar, aminated plant biomass, aminated silica, slag, and fly ash. As already mentioned, this could be due to the structure of the orthophosphate ion, which can form more hydrogen bonds with hydrogen, oxygen and nitrogen atoms of the functional groups of the sorbent compared to the nitrate ion.

The sorption efficiency of orthophosphates and nitrates in wastewater is generally lower than in solutions based on distilled water. This is due to the competition of the nutrient ions (e.g., H_2_PO_4_^−^, HPO_4_^2−^, and NO_3_^−^) with other anions in the solution (e.g., SO_4_^2−^, Cl^−^, F^−^, and Br^−^) for the active sites of the sorbents. This is confirmed by our research on wastewater treatment with chitosan hydrogels [25].

Taking into account the main mechanisms of sorption of orthophosphates and nitrates to sorbents, including their electrostatic interactions with ionized functional groups and the formation of hydrogen bonds between them, selective sorption of the selected nutrient seems practically impossible.

Chitin flakes used in the treatment of greenhouse effluents may be regenerated by, e.g., subjecting them to a desorption process. Presumably, nutrient release from CH would be extensive if the sorbent had been placed in an alkaline solution (pH > 10). Alternatively, spent CH can be used in agriculture as a phosphate-rich and nitrate-rich fertilizer component that also improves soil structure. They could also be utilized as a component in mats used for fertigation in soilless plant cultivation. However, this requires further research.

## 3. Materials

### 3.1. Chitin Flakes (CH)

Chitin (CH) flakes from snow crab shells were obtained from Biolog Heppe^®®^ GmbH (Landsberg, Germany). A fraction of flakes with a diameter of 2–3 mm was used for the study. The BET area reported for CH was 2.486 m^2^/g. The pore diameter of the material averaged 1.84 nm, indicating its microporosity. The total pore volume of CH was 0.00115 cm^3^/g. The results of the CH surface and porosity analyses (Figures S1 and S2) are provided in the Supplementary Data.

The characteristics of the FTIR spectrum as well as the SEM analysis used in the study of the chitin flakes were presented in our previous article [46]. SEM photos of CH are also included in the Supplementary Materials (Figures S3 and S4).

### 3.2. Greenhouse Wastewater (GW)

The greenhouse wastewater (GW) used in the study originated from the soilless cultivation of tomatoes in greenhouses in the Warmia–Masuria Voivodeship (Poland). Tomatoes of the following varieties were grown in the greenhouses: Growdena F1, Listell F1 and Torero F1. The plants were grown on a coconut fiber substrate. The nutrient solution for tomatoes was prepared by adding calcium nitrate, saltpeter, potassium phosphate, potassium sulfate, magnesium sulfate, iron chelate and a ready-made mixture of trace elements to tap water. The dosage of the individual ingredients was adapted to the developmental stage of the plants. The greenhouse wastewater (GW) from the cultivation was averaged and collected in IBC tanks protected from sunlight from 1 September 2022 to 31 October 2022. The most important GW parameters are listed in Table 5.

### 3.3. Chemical Reagents

The following chemical reagents were used in the research:

- Sodium hydroxide (NaOH) ≥ 98.0% (powder)—used to correct the pH of wastewater;
- Hydrochloric acid (HCl) 37.0%—used to correct the pH of wastewater;
- Buffer solutions for calibrating the pH meter (pH 4 ± 0.05/pH 7 ± 0.05/pH 10 ± 0.05).

All chemical reagents used were purchased from Sigma-Aldrich (St. Louis, MO, USA) and were of analytical purity (a.p.) or higher.

### 3.4. Laboratory Equipment

The following chemical reagents were used in the research:

- EX2202 precision balance (OHAUS, Nänikon, Switzerland)—for preparing solutions and weighing the sorbent;
- HI 221 pH-meter (Hanna Instruments, Woonsocket, RI, USA)—for the measurement and correction of the solutions’ pH;
- Laboratory shaker SK-71 (JEIO TECH, Daejeon, Republic of Korea)—(for the process of sorption);
- Multi-Channel Stirrer MS-53M (JEIO TECH, Daejeon, Republic of Korea)—for the process of sorption.

## 4. Methodology

### 4.1. Research on pH Correction Influence on GW Composition

Amounts of 800 mL of GW were added to 11 beakers (1000 mL capacity) using a precision balance. The pH was then adjusted in 10 beakers to the following values: pH 2.0, 3.0, 4.0, 5.0, 6.0, 7.0, 8.0, 9.0, 10.0, and 11.0. The pH was corrected by dosing small amounts of HCl or NaOH (0.1/1.0 M) into the GW while continuously measuring its value. After pH correction, the beakers with the solutions were left to stand for 60 min (during this time, the contents of the beaker were not stirred). Afterward, samples were taken from the beakers to determine the concentration of selected elements (P-PO_4_, N, S, Ca, Mg) in the solutions. The conductivity of the wastewater after pH correction was measured as well.

### 4.2. Research on pH Influence on the Efficiency of Nutrient Sorption from GW

Amounts of 1.250 g of CH were weighed into 12 Erlenmeyer flasks (1000 mL capacity). Then, 250 mL of GW (with a pH of 2.0, 3.0, 4.0, 5.0, 6.0, 7.0, 8.0, 9.0, 10.0 and 11.0) were added to the flasks. GW with its natural pH—(pH 5.4) was added to the last flask). The flasks were placed on a multi-station shaker (150 rpm, oscillation amplitude 30 mm). After 60 min of sorption, samples were taken from the flasks to determine the concentration of the components remaining in the solutions. The pH value and the conductivity of the wastewater after sorption were measured as well.

### 4.3. Determination of the pH_PZC_ of CH

An amount of 0.01 M solution of NaCl with the following pHs: pH 2, pH 3, pH 4, pH 5, pH 6, pH 7, pH 8, pH 9, pH 10, and pH 11 (pH was adjusted with aqueous solutions of HCl and NaOH) were prepared in 10 measuring flasks (250 mL capacity). Then, 1.250 g portions of CH were introduced to 10 conical flasks (1000 mL capacity), and the next 250 mL portions of the earlier prepared sodium chloride solutions (with pH 2–11) were added to the flasks. The flasks were protected with a parafilm and placed on a multi-station shaker (150 rpm, oscillation amplitude 30 mm). The pH of the solutions in the flasks was measured after 240 min. The changes in pH values of the solutions (pH_E_-pH_0_, i.e., pH value of the solution measured after 240 min of contact with the sorbent minus the initial pH of the solution) were provided in the figure in the function of initial pH. The initial pH at which pH_E_-pH_0_ reached “0” indicated the pH_PZC_ of the sorbent.

### 4.4. Research on the Kinetics of Nutrient Sorption from GW

An amount of 4.000/40.000 g of CH was weighed into beakers (1000 mL capacity), and then 800 mL of GW with the optimum sorption pH for phosphates (determined based on analyses described in Section 4.2) were added to the beakers. Then, the beakers were placed on a multi-station magnetic stirrer (200 rpm, Teflon-coated stirrer 50 × 8 mm). After the specified times (0, 10, 20, 30, 45, 60, 90, 120, 150, 180, 210, 240, and 300 min), samples of the solutions (5 mL) were collected from the beakers using an automatic pipette to the previously prepared test tubes.

### 4.5. Research on CH Dose Influence on the Sorption Efficiency of P-PO_4_ and N-NO_3_ from GW

CH (1.600, 2.400, 4.000, 8.000, 12.000,16.000, 20.000, 24.000, 28.000, 32.000, 36.000, 40.000, 48.000, 64.000, and 80.000 g) was introduced into 15 beakers (1000 mL capacity). Amounts of 800 mL of GW with the optimum pH value for phosphorus sorption (determined on the basis of analyses described in Section 4.2) were added to the beakers. The beakers were placed on a multi-station stirrer (200 rpm, stirrer 50 × 8 mm) until sorption equilibrium was reached (determined on the basis of analyses described in Section 4.4). After the specified time, samples (10 mL) were taken from the solutions for the analysis of nutrient concentrations.

#### Notes to Section 4.1, Section 4.2, Section 4.3, Section 4.4 and Section 4.5

- The preparation of the solutions and the weighing of the sorbents in laboratory flasks or beakers were carried out using a precision balance with an accuracy of 0.001 g.
- Beakers with GW were weighed before and after pH correction in order to take into account the change in volume of the GW sample caused by the addition of acidifying/alkalizing agents later in the calculations.
- The mixing parameters, set on a shaker or a multi-station mixer, ensured the distribution of CH throughout the GW volume.
- Nutrient concentrations in the GW were determined according to Polish standards: for P-PO_4_—PN-EN 6878:2006 and for N-NO_3_—PN-73/C-04576/06.
- The concentrations of other substances (sulfates, calcium and magnesium ions) were determined using HACH cell tests (HACH LANGE Sp. z o. o., Wrocław, Poland).
- When selecting the optimum sorption pH value, the effectiveness of phosphorus binding was taken into account due to the primary economic importance of this element.
- All analytical series were carried out in triplicate.
- During analyses, the temperature in the laboratory was kept constant at 20 °C.

### 4.6. Computation Methods

The amount of nutrients sorbed by CH was calculated according to Formula (1):

(1) $$Q_{S}=(C_{0}-C_{S})\times \frac{V}{m}$$

*Q_S_*—mass of sorbed nutrient [mg/g];
*C*_0_—initial concentration of nutrient in GW [mg/L];
*C_S_*—concentration of nutrient after sorption [mg/L];
*V*—volume of GW [L];
*m*—mass of CH [g].

The kinetics of nutrient sorption to CH was described by pseudo-first-order (2), pseudo-second-order (3) and intraparticle diffusion (4) models.

(2) $$Q=q_{e}\times (1-e^{\left(-k_{1}\times t\right)})$$

(3) $$Q=\frac{\left(k_{2}\times {q_{e}}^{2}\times t\right)}{\left(1+k_{2}\times q_{e}\times t\right)}$$

(4) $$Q=k_{id}\times t^{0.5}$$

*Q*—instantaneous value of sorbed nutrient [mg/g];
*q_e_*—the amount of nutrient sorbed at the equilibrium state [mg/g];
*t*—time of sorption [min];
*k*_1_—pseudo-first-order adsorption rate constant [1/min];
*k*_2_—pseudo-second-order adsorption rate constant [g/(mg·min)];
*k_id_*—intraparticle diffusion model adsorption rate constant [mg/(g·min^0.5^)].

Experimental data from studies on the maximum sorption capacity of CH toward P-PO_4_ and N-NO_3_ were described by the two best-known sorption models: the Langmuir isotherm (5) and the Freundlich isotherm (6).

(5) $$Q_{S}=\frac{\left(Q_{max}\times K_{C}\times C\right)}{\left(1+K_{C}\times C\right)}$$

(6) $$Q_{S}=K\times C^{\frac{1}{n}}$$

*Q_S_*—mass of sorbed nutrient [mg/g];
*Q_max_*—maximum sorption capacity in Langmuir equation [mg/g];
*K_C_*—constant in Langmuir equation [L/mg];
*K*—the equilibrium sorption constant in the Freundlich model;
*n*—Freundlich equilibrium constant;
*C*—concentration of the dye remaining in the solution [mg/L].

## 5. Conclusions

The present study results show that it is possible to use chitin flakes to sift orthophosphates and nitrates from greenhouse effluents, but their efficiency as sorbents is relatively low. A sorbent dose of 40 g CH/L was required to remove 90% of P-PO_4_ from GW by sorption. This dose also ensured the removal of 5.7% of N-NO_3_ from the wastewater. The maximum sorption capacity of CH towards P-PO_4_ and N-NO_3_ contained in GW was 3.20 mg/g and 3.04 mg/g, respectively. A review of the literature data shows that chitin sorbents are many times more efficient in treating the same wastewater.

The efficiency of sorption of nutrients from wastewater is generally lower than from the distilled water-based solutions. This is due to the presence of other anions in the wastewater, e.g., chloride and sulfate, which compete with orthophosphates and nitrates for the active centers of the sorbent.

CH showed preferential sorption of P-PO_4_ compared to N-NO_3_. This could be due to the structure of the orthophosphate ion, which is able to form a larger number of hydrogen bonds with the functional groups of the sorbent compared to the nitrate ion. However, selective binding of individual nutrients is impossible.

The efficiency of nutrient sorption to CH from GW depended largely on the sorption pH value and was the highest at pH 4 and pH 2 in the case of orthophosphates and nitrates, respectively.

CH can modify the pH of greenhouse effluents. During sorption, the system always strives for a pH value that is close to the pH value of the sorbent used (for CH pH_PZC_ = 7.1). The higher the dose of sorbent used, the stronger this effect is.

The equilibrium time of both nutrients sorption from GW to CH was between 150 and 180 min and was shorter at higher sorbent doses. The sorption of P-PO_4_ and N-NO_3_ onto CH occurred in three main phases that differed in intensity and duration.

Effective removal of orthophosphates from GW can be achieved by simply correcting the pH of the wastewater (e.g., with NaOH). Under alkaline conditions (pH > 7), the orthophosphate ions are precipitated with the calcium and magnesium ions present in the wastewater. Increasing the pH of greenhouse wastewater to pH 8 and pH 9, for example, reduced the P-PO_4_ concentration in the system by 93% and 98%, respectively. Due to the inability to precipitate nitrate ions with Ca^2+^ and Mg^2+^ ions, pH correction had no effect on the N-NO_3_ concentration in the GW.

It was found that Ca^2+^ and Mg^2+^ ions were practically not sorbed to CH, regardless of the pH of the GW. However, as mentioned, calcium and magnesium ions can be removed from the system at pH values > 7 by precipitation with the orthophosphates in the wastewater. In the case of calcium ions, precipitation with sulfates is also possible. At higher pH values (pH > 9), some Ca^2+^ and Mg^2+^ ions can also be precipitated from the wastewater as hydroxides.

The sorption of sulfates from GW on CH is only feasible at very low pH values (pH 2–3). The removal of S-SO_4_ from wastewater is also possible by precipitation with calcium ions under alkaline conditions (pH > 7).

## Figures and Tables

**Table 1:** Kinetic parameters of phosphate (P-PO_4_) and nitrate (N-NO_3_) sorption on CH, determined from the pseudo-first order and pseudo-second order models (based on the average of three measurements) + sorption equilibrium time). Sorbent dose—5 g/L; initial pH 4; temp. 20 °C.

Sorbate	CH Dose	Pseudo-First Order Model			Pseudo-Second Order Model			Exp. Data	Equil. Time
		*k*_1_	*q_e, cal._*	*R* ^2^	*k*_2_	*q_e, cal._*	*R* ^2^	*q_e, exp._*	
	[g/L]	[1/min]	[mg/g]	-	[g/mg·min]	[mg/g]	-	[mg/g]	[min]
P-PO_4_	5	0.0437	2.63	0.9370	0.0222	2.90	0.9799	2.77	180
	50	0.1651	1.07	0.9326	0.2297	1.13	0.9832	1.14	150
N-NO_3_	5	0.0494	2.54	0.9707	0.0256	2.79	0.9943	2.64	180
	50	0.0809	0.61	0.9698	0.1892	0.65	0.9952	0.63	150

**Table 2:** The rate constants of phosphate and nitrate diffusion were determined from a simplified intraparticle diffusion model. Sorbent dose—5/50 g/L; initial pH 4; temp. 20 °C.

Sorbate	CH Dose	Phase 1			Phase 2			Phase 3		
		*k_d1_*	Dur. Time	*R* ^2^	*k_d2_*	Dur. Time	*R* ^2^	*k_d3_*	Dur. Time	*R* ^2^
	[g/L]	*	[min]	-	*	[min]	-	*	[min]	-
P-PO_4_	5	0.4112	10	0.(9)	0.1973	50	0.9713	0.0758	120	0.9955
	50	0.3200	5	0.(9)	0.0583	40	0.9995	0.0221	105	0.9925
N-NO_3_	5	0.3664	20	0.9954	0.1743	70	0.9732	0.0329	90	0.8238
	50	0.1105	10	0.(9)	0.0516	35	0.9915	0.0115	105	0.9077

*—Unit for parameters *k_d1_*, *k_d2_* and *k_d3_*—[mg/(g·min^0.5^)].

**Table 3:** Constants determined from Langmuir and Freundlich models. Initial pH 4; sorption time—180 min, temp. 20 °C.

Sorbate	Langmuir Model			Freundlich Model		
	*Q_max_*	*K_C_*	*R* ^2^	*k*	*n*	*R* ^2^
	[mg/g]	[L/mg]	-	-	-	-
P-PO_4_	3.20	0.112	0.9870	0.707	0.347	0.9359
N-NO_3_	3.04	0.058	0.9627	0.875	0.352	0.9523

**Table 4:** Comparison of the sorption efficiency of nutrients from wastewater and mixtures using different unconventional sorbents.

Type of the Sorbent	Type of Solution	Sorbate	*Q_max_* (mg/g)	pH	Time [h]	Temp.	Source
Chitosan hydrogel beads modified with epichlorohydrin	deionized water + sodium phosphate	P-PO_4_	139.4	3	2	22	[30]
	deionized water + sodium nitrate	N-NO_3_	38.47	3	2	22	[37]
	equimolar mixture of nutrients based on deionized water	mixture P-PO_4_; N-NO_2_; N-NO_3_	62.01 (total N + P) 38.22; 13.09; 10.70	3	2	22	[23]
	greenhouse wastewater (60.8 mg P-PO_4_/L; 621 mg N-NO_3_/L)	mixture P-PO_4_; N-NO_3_	60.9 (total N + P) 55.9; 5.0	2	3	22	[25]
Biochar-MgAl LDH Nanocomposites	synthetic wastewater (50 mg P-PO_4_/L; 50 mg N-NO_3_/L)	mixture P-PO_4_; N-NO_3_	102.95 (total N + P) 73.69; 28.26	6	24	25	[39]
Chitosan resin	synthetic wastewater (100 mg P-PO_4_/L; 100 mg N-NO_3_/L)	mixture P-PO_4_; N-NO_3_	85.93 (total N + P) 37.08; 48.85	3	1	30	[40]
Chitosan hydrogel beads (unmodified)	deionized water + sodium phosphate	P-PO_4_	44.40	4	2	22	[30]
	deionized water + sodium nitrate	N-NO_3_	12.71	2	4	22	[37]
	equimolar mixture of nutrients based on deionized water	mixture P-PO_4_; N-NO_2_; N-NO_3_	25.84 (total N + P) 15.72; 5.22; 4.90;	4	1	22	[29]
	greenhouse wastewater (60.8 mg P-PO_4_/L; 621 mg N-NO_3_/L)	mixture P-PO_4_; N-NO_3_;	24.99 (total N + P) 5.3419.65	4	3	22	[25]
Aminated wheat straw	synthetic wastewater (50 mg P-PO_4_/L; 60 mg N-NO_3_/L)	mixture P-PO_4_; N-NO_3_	26.83 (total N + P) 14.9 111.92	3	4	20	[41]
Aminated silica MCM-48	synthetic wastewater (700 mg P-PO_4_/L; 700 mg N-NO_3_/L)	mixture P-PO_4_; N-NO_3_	21.38 (total N + P) 13.52 7.86		4	25	[42]
Waste residue from alum manufacturing (quartz, kaolin, aluminum Hydroxide)	synthetic wastewater (19.3 mg P-PO_4_/L; 5.1 mg N-NO_3_/L)	mixture P-PO_4_; N-NO_3_	13.17 (total N + P) 13.10; 0.07	4–8	1.5	25	[43]
Chitosan flakes	deionized water + potassium phosphate	P-PO_4_	6.64	4	0.66	22	[28]
Chitin flakes (CH)	greenhouse wastewater (66.2 mg P-PO_4/_L; 566 mg N-NO_3_/L)	mixture P-PO_4_; N-NO_3_	6.24 (total N + P) 3.20; 3.04	4	3.0	20	This work
Slag	industrial wastewater (40 mg P-PO_4_/L; 32 mg N-NO_3_/L)	mixture P-PO_4_; N-NO_3_;	3.28 (total N + P) 2.51; 0.77;	5	0.5	27	[44]
Fly ash	industrial wastewater (40 mg P-PO_4_/L; 32 mg N-NO_3_/L)	mixture P-PO_4_; N-NO_3_	2.76 (total N + P) 2.53; 0.23;	7	0.5	27	[44]
Bagasse (agricultural residues)	mixture of nutrients based on deionized water (38 mg P-PO_4_/L; 37 mg N-NO_2_/L; 37 mg N-NO_3_/L)	mixture P-PO_4_; N-NO_2_; N-NO_3_	0.72 (total N + P) 0.16; 0.31; 0.25;	6.5	24	30	[45]

**Table 5:** Parameters of the greenhouse wastewater (GW) used in the study.

Component of GW	P-PO_4_ [mg/L]	N-NO_3_ [mg/L]	S-SO_4_ [mg/L]	Cl^−^ [mg/L]	Ca^2+^ [mg/L]	Mg^2+^ [mg/L]	K^+^ [mg/L]	Hardness [°dH]	pH
Content	66.2	566.0	456.0	13.7	721.0	230.0	980.6	11.3	5.4

## Data Availability

The data presented in this study are available on request from the corresponding author.

## Supplementary Materials

The following supporting information can be downloaded at: https://www.mdpi.com/article/10.3390/molecules29061289/s1, CH surface and porosity test report; Figure S1. N_2_ adsorption isotherm on the CH surface; Figure S2. Incremental Pore Volume vs. Pore Width—CH Surface analysis; Figure S3. SEM images of CH—part 1; Figure S4. SEM images of CH—part 2.
