# Supplementary material for: The Use of Chitin for the Removal of Nitrates and Orthophosphates from Greenhouse Wastewater
Source: Molecules. 2024 Mar 14;29(6):1289. doi: 10.3390/molecules29061289 (PMC10974353; doi:10.3390/molecules29061289)
Supplement: Supplementary file 1 [file molecules-29-01289-s001.zip › molecules-2909020-supplementary.pdf]

Supplementary materials:

CH surface and porosity test report

Summary Report

Surface Area

Single point surface area at p/p° = 0.250270095: 2.4078 m²/g

BET Surface Area: 2.4859 m²/g

Pore Volume

Single point adsorption total pore volume of pores
less than 2.3143 nm diameter at p/p° = 0.250270095: 0.001141 cm³/g

Pore Size

Adsorption average pore width (4V/A by BET): 1.83625 nm

DFT Pore Size

Volume in Pores	<	1.416 nm	:	0.00000 cm³/g
Total Volume in Pores	<=	38.734 nm	:	0.00115 cm³/g
Area in Pores	>	38.734 nm	:	0.000 m²/g
Total Area in Pores	>=	1.416 nm	:	1.937 m²/g

Started: 2022-10-26 9:54:00
Completed: 2022-10-26 12:11:47
Report Time: 2022-10-28 10:32:44
Sample Mass: 0.1543 g
Equilibration Interval: 5 s
Sample Density: 1.000 g/cm³

Analysis Adsorptive: N2
Analysis Bath Temp.: 77.370 K
Thermal Correction: No
Cold Free Space: -0.8919 cm³ Measured
Low Pressure Dose: None
Automatic Degas: No

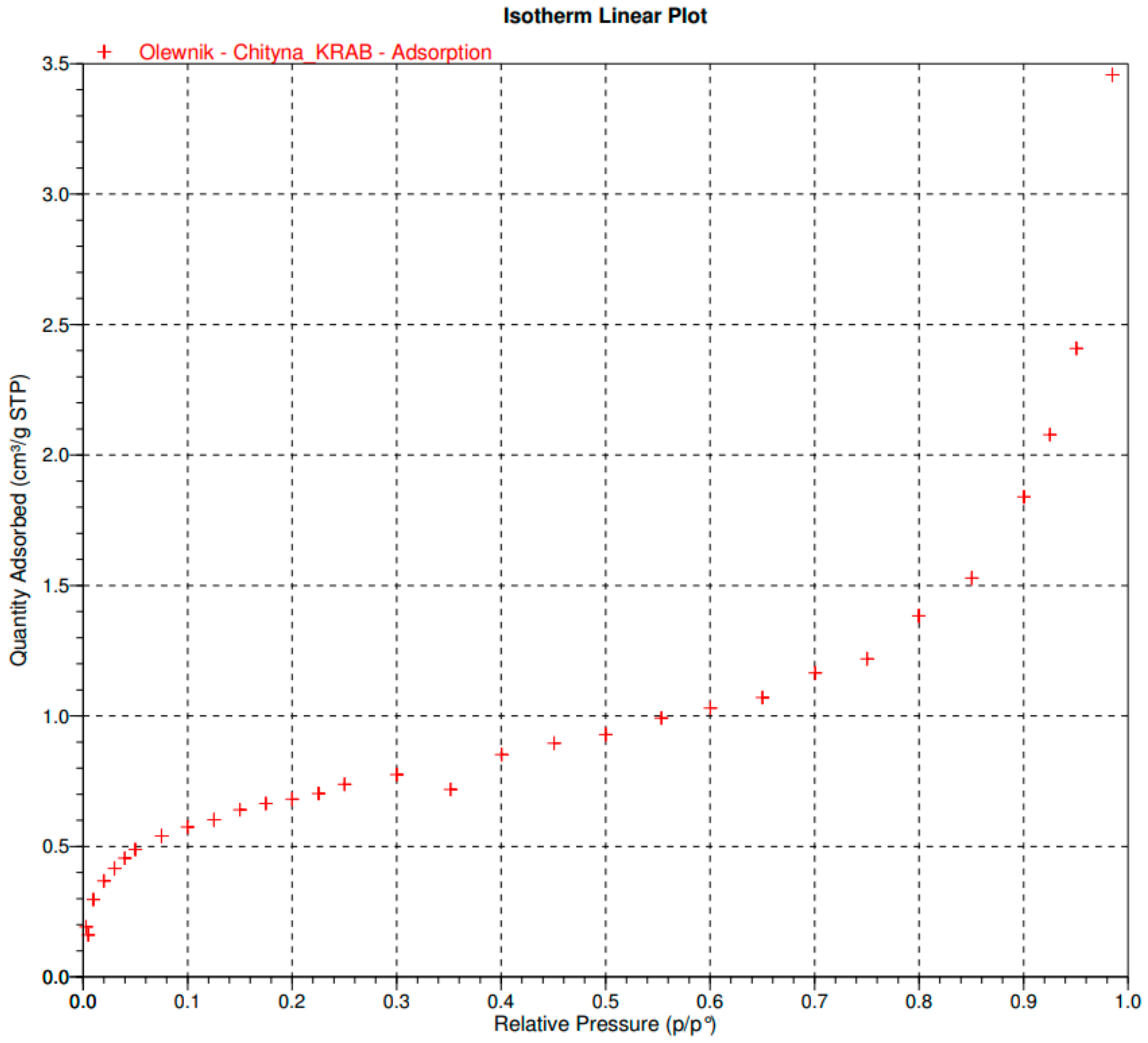

Figure S1. N2 adsorption isotherm on the CH surface

Started: 2022-10-26 9:54:00
Completed: 2022-10-26 12:11:47
Report Time: 2022-10-28 10:32:44
Sample Mass: 0.1543 g
Equilibration Interval: 5 s
Sample Density: 1.000 g/cm³

Analysis Adsorptive: N_2
Analysis Bath Temp.: 77.370 K
Thermal Correction: No
Cold Free Space: -0.8919 cm³ Measured
Low Pressure Dose: None
Automatic Degas: No

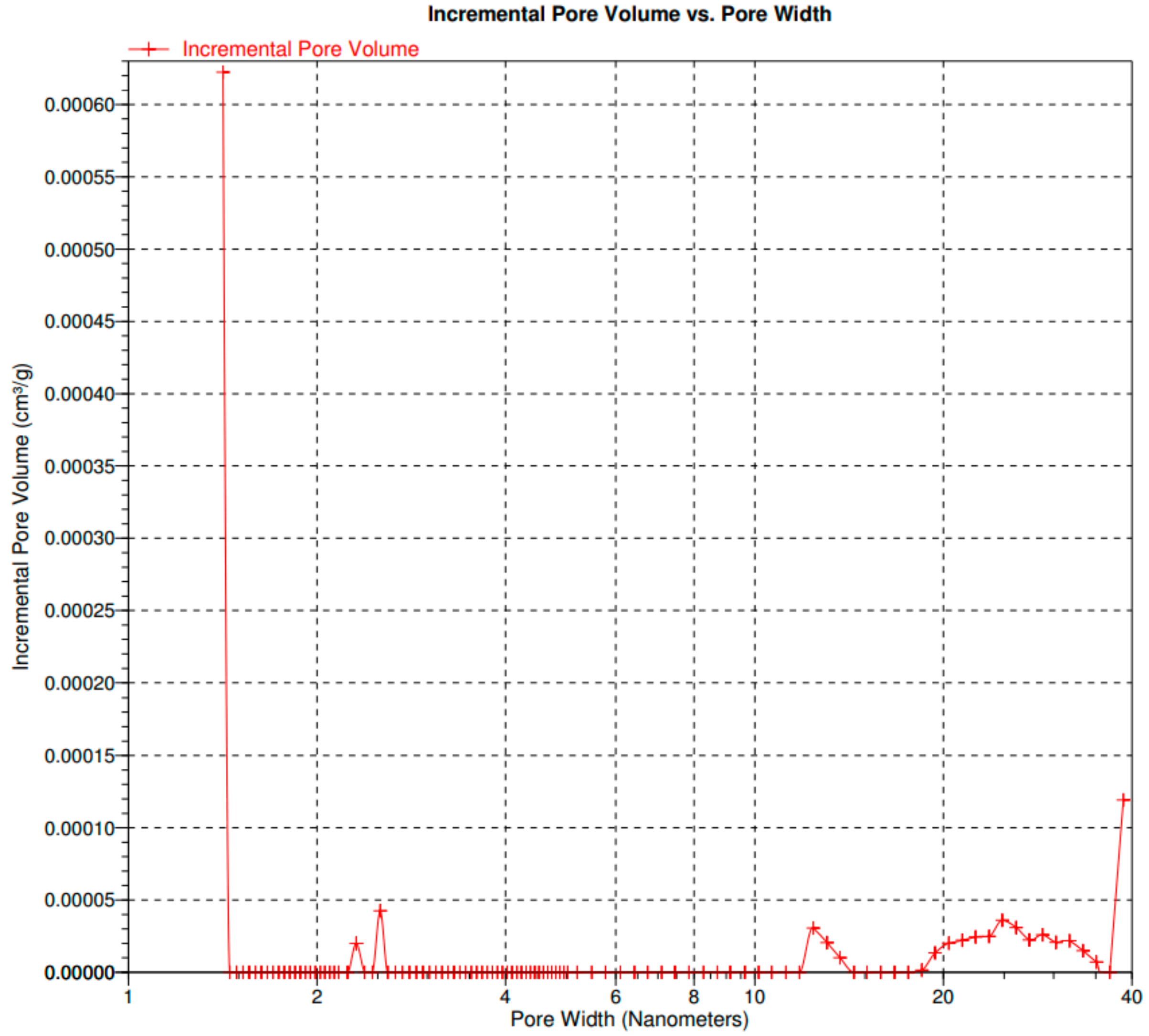

Figure S2. Incremental Pore Volume vs. Pore Width – CH Surface analysis

SEM images of CH

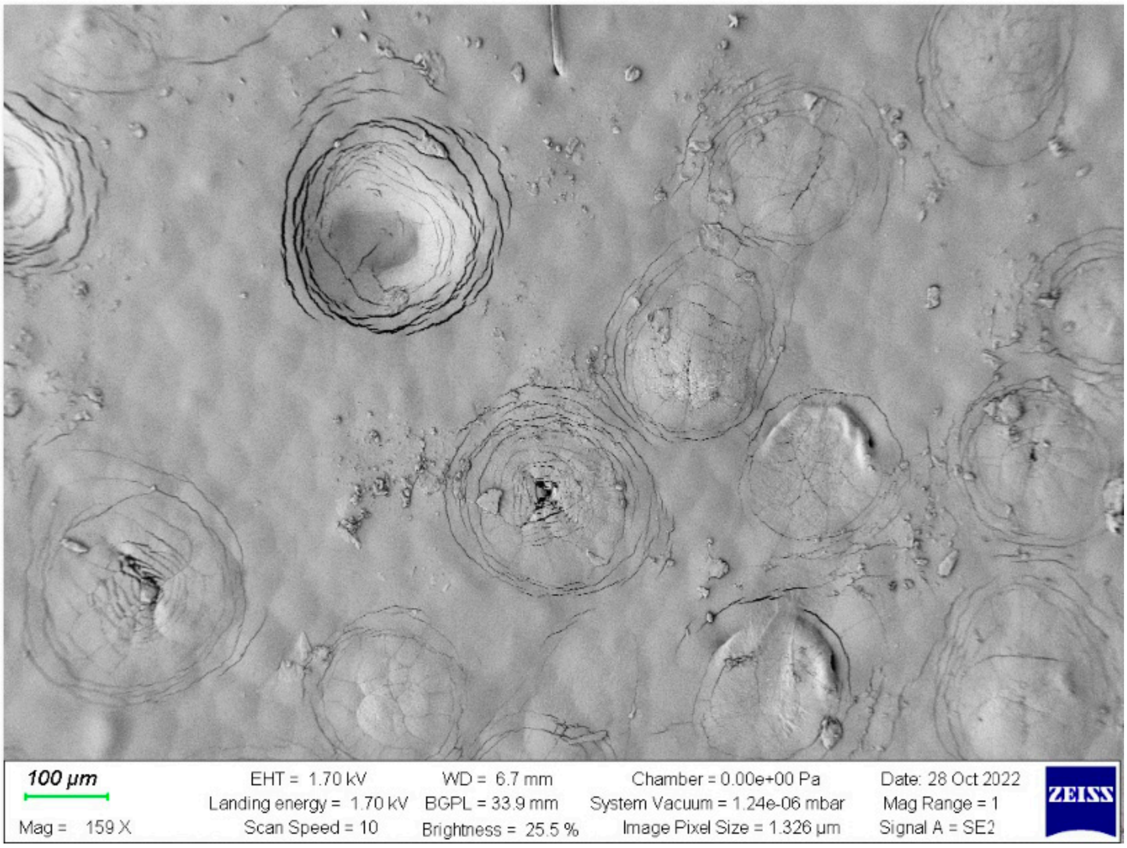

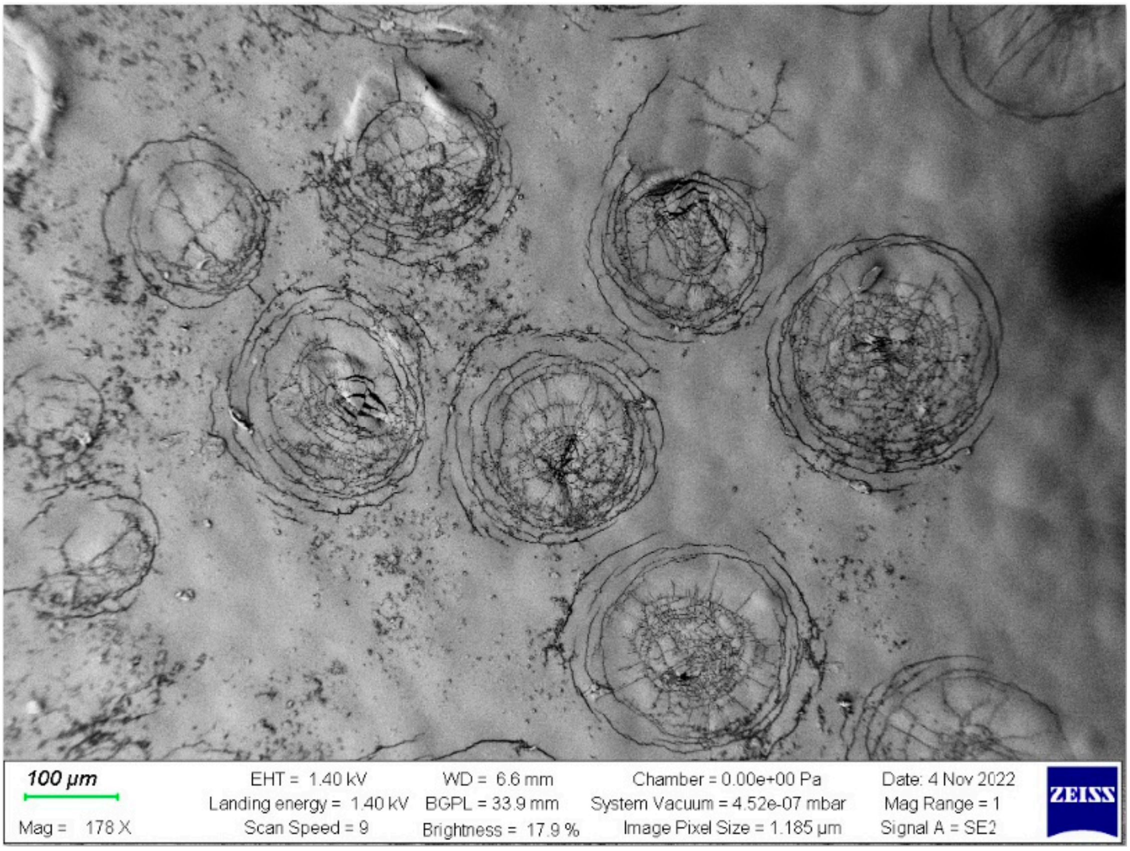

Figure S3. SEM images of CH – part 1

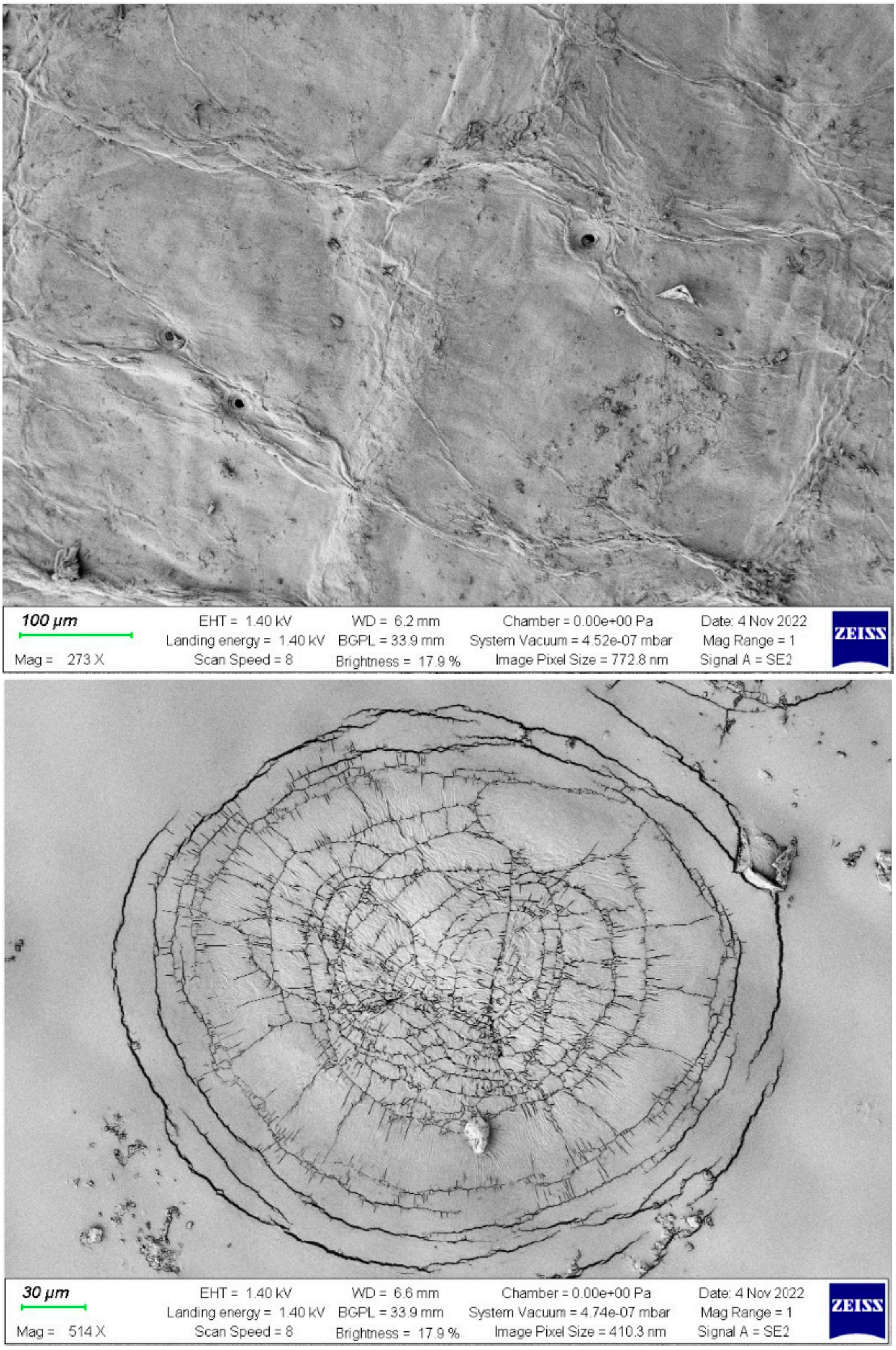

Figure S4. SEM images of CH – part 2
